# From the Ground Up: Global Nitrous Oxide Sources are Constrained by Stable Isotope Values

**DOI:** 10.1371/journal.pone.0118954

**Published:** 2015-03-26

**Authors:** David M. Snider, Jason J. Venkiteswaran, Sherry L. Schiff, John Spoelstra

**Affiliations:** 1 National Water Research Institute, Canada Centre for Inland Waters, Environment Canada, Burlington, ON, L7R 4A6, Canada; 2 Department of Geography and Environmental Studies, Wilfrid Laurier University, Waterloo, ON, N2L 3C5, Canada; 3 Department of Earth and Environmental Sciences, University of Waterloo, Waterloo, ON, N2L 3G1, Canada; North Carolina State University, UNITED STATES

## Abstract

Rising concentrations of nitrous oxide (N_2_O) in the atmosphere are causing widespread concern because this trace gas plays a key role in the destruction of stratospheric ozone and it is a strong greenhouse gas. The successful mitigation of N_2_O emissions requires a solid understanding of the relative importance of all N_2_O sources and sinks. Stable isotope ratio measurements (δ^15^N-N_2_O and δ^18^O-N_2_O), including the intramolecular distribution of ^15^N (site preference), are one way to track different sources if they are isotopically distinct. ‘Top-down’ isotope mass-balance studies have had limited success balancing the global N_2_O budget thus far because the isotopic signatures of soil, freshwater, and marine sources are poorly constrained and a comprehensive analysis of global N_2_O stable isotope measurements has not been done. Here we used a robust analysis of all available *in situ* measurements to define key global N_2_O sources. We showed that the marine source is isotopically distinct from soil and freshwater N_2_O (the continental source). Further, the global average source (sum of all natural and anthropogenic sources) is largely controlled by soils and freshwaters. These findings substantiate past modelling studies that relied on several assumptions about the global N_2_O cycle. Finally, a two-box-model and a Bayesian isotope mixing model revealed marine and continental N_2_O sources have relative contributions of 24–26% and 74–76% to the total, respectively. Further, the Bayesian modeling exercise indicated the N_2_O flux from freshwaters may be much larger than currently thought.

## Introduction

Since the advent of the Haber-Bosch process one century ago, humans have vastly perturbed the global nitrogen (N) cycle. Current anthropogenic activities contribute 51% of the total N fixed worldwide (210 of 413 Tg N yr^−1^) [1]. One negative consequence of this is an increase in atmospheric nitrous oxide (N_2_O) [2], a long-lived trace gas that contributes to climate warming and the destruction of stratospheric ozone [3]. The current concentration of N_2_O in the troposphere is 325 parts per billion (ppb) [4]. Future concentrations of atmospheric N_2_O are difficult to predict, yet this information is an essential input parameter for global climate change models. Further, both the prediction and mitigation of N_2_O concentrations depend on an accurate understanding of the emissions from key N_2_O sources.

Most emissions of N_2_O (natural and anthropogenic) occur from terrestrial, freshwater, and marine environments, where N compounds are processed by nitrifying and denitrifying microorganisms. These processes account for ∼89% of the total annual N_2_O emissions, or almost 16 Teragrams (Tg = 10^12^ g) N/year [5]. However, scientists’ best estimates of the N_2_O budget are still highly uncertain. The most recent Intergovernmental Panel on Climate Change Assessment Report (IPCC-AR5) reveals wide ranges in the relative uncertainty of many individual N_2_O sources. In addition, the uncertainty on the annual cumulative emissions of N_2_O for 2006 from natural soils, oceans, rivers, estuaries, coastal zones, and agriculture combined ranged between 6.9–26.1 Tg N [5].

**Fig 1:**
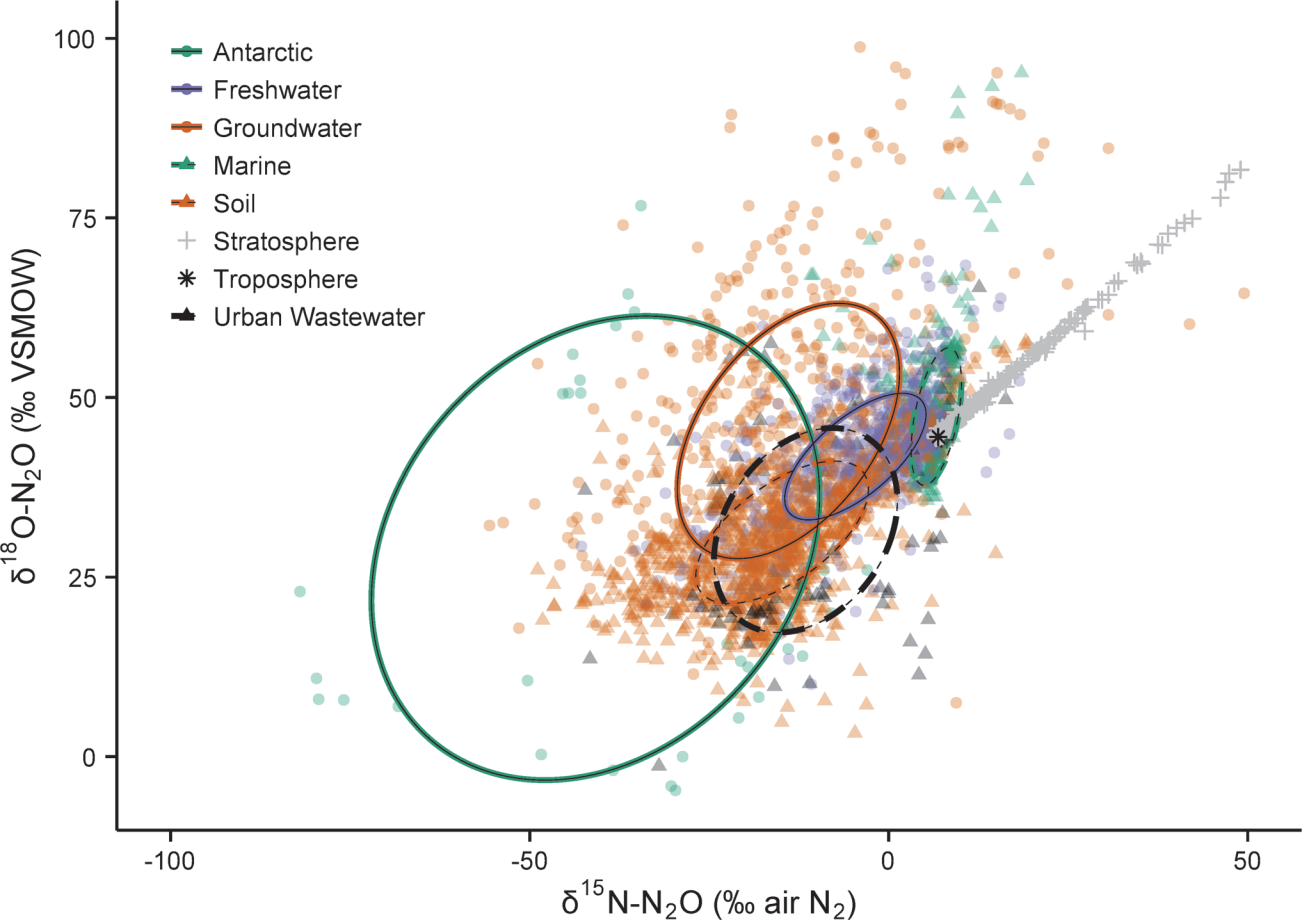
Global N_2_O isotope measurements from atmospheric, marine, and terrestrial samples. All the data compiled in this study that fit within the axis ranges shown are plotted here. Each point represents one measurement, or in a few cases a reported average value, and is two-thirds transparent to allow the density of data to be displayed. Standard ellipses encompass ∼40% of the data (Fig. 2) and are shown for the six non-atmospheric categories. Most data fall to the left of the current tropospheric value. Stratospheric data falls along a line (δ^18^O = 0.89 × δ^15^N + 38.4) (R^2^ = 0.999) that originates from the tropospheric value, and is caused by isotopic fractionation during N_2_O destruction [13].

The clear separation and accounting of individual N_2_O sources remains challenging, but is essential if we are to make meaningful reductions in emissions. Measurements of stable isotope ratios (δ^15^N-N_2_O and δ^18^O-N_2_O) and the intramolecular site preference (SP) of ^15^N are one way to track sources *if* they are isotopically distinct. Several accounts of the global N_2_O budget have used ‘top-down’ isotope mass-balance models to estimate the strength and isotopic composition of anthropogenic and natural N_2_O sources [2,6–11]. In this approach, changes in atmospheric N_2_O over time are modelled by comparing our modern-day atmosphere (a mixture of post-industrial, anthropogenic N_2_O and natural N_2_O) to relic air trapped in glacial firn and ice. All these studies have assumed that soils are the main source of post-industrial N_2_O because its calculated isotopic composition was most similar to a limited body of published soil N_2_O measurements. Yet we do not have a clear synthesis of the isotopic character of individual N_2_O sources. For example, freshwaters and estuaries may contribute up to 25% of anthropogenic N_2_O emissions [5], but prior to 2009 there was only one publication reporting freshwater δ^15^N-N_2_O and δ^18^O-N_2_O values [12] (S1 Dataset). In reality, there is extreme variation in the measured values of δ^15^N-N_2_O and δ^18^O-N_2_O (Fig. 1), and no systematic examination of individual sources has occurred.

In this paper, we use a ‘bottom-up’ approach to define key N_2_O sources and demonstrate that their global average δ^15^N and δ^18^O values are isotopically unique. Further we use these *in situ* N_2_O isotope data to substantiate what ‘top-down’ global atmospheric models have predicted; soils, and not marine or freshwater ecosystems, are the main source of rising atmospheric N_2_O levels.

## Methods

We mined 1920 data points from 52 studies that measured *in situ* δ^15^N-N_2_O and δ^18^O-N_2_O in atmospheric, terrestrial and marine systems from 1987 to present [2,10–60]. If the published data was not tabulated, we used the software ‘g3data’ (*http://www.frantz.fi/software/g3data.php*) to extract data from figures [61]. The accuracy of our method was tested by plotting a subset of data from Well *et al*. [51], re-extracting it, and then comparing it to the original values. The mean (min/max) difference (‰) was 0.06 (0.00/0.13) for δ^15^N and 0.02 (0.00/0.07) for δ^18^O. This represents a worst-case accuracy of our ability to extract data from figures because the test data had an unusually wide range (−80 to +120‰ for δ^15^N, and 0 to +120‰ for δ^18^O; *n* = 53) and all other published graphs had much smaller scales. Values of δ^18^O-N_2_O reported vs. atmospheric O_2_ were converted to δ^18^O-N_2_O vs. Vienna Standard Mean Ocean Water (VSMOW) according to Kim and Craig [19].

Twenty-seven studies also measured the intramolecular distribution of ^15^N in the linear NNO molecule (780 data points) and these data are provided in the supplementary datasets (S1 Dataset and S2 Dataset). This difference between the central (δ^15^N^α^) and terminal (δ^15^N^β^) ^15^N enrichment is often expressed as the site preference (SP). This parameter is thought to be a unique indicator of the microbial pathway that produces N_2_O and not to be affected by variations in the isotopic ratios of substrates. Recently, this idea has been called into question by Yang *et al*. [62], who showed that SP can vary depending on the growth conditions of microbial cultures. Regardless, if the SP of different global sources is unique it can be used in conjunction with traditional measures of δ^15^N-N_2_O and δ^18^O-N_2_O values to separate sources in three-dimensional isotope space.

To this compendium of published data we added 1367 new *in situ* δ^15^N-N_2_O and δ^18^O-N_2_O data from 16 sites across Ontario and New Brunswick, Canada (S1 Dataset). Soil pore gas and static flux chambers were sampled at four Ontario sites. Urban wastewater treatment plants, streams, rivers, and agricultural drainage tile outlets were sampled across four watersheds in Ontario and New Brunswick. Groundwaters were sampled from numerous domestic and monitoring wells (some multi-level) distributed across nine research sites in Ontario and New Brunswick.

Liquid samples were stripped of N_2_O using an off-line purge-and-trap system described in Baulch *et al*. [35]. With the exception of samples from one location (ERS) that were analyzed at UC Davis-SIF, all analyses occurred at the University of Waterloo on an IsoPrime isotope ratio mass spectrometer (IRMS) with a TraceGas pre-concentrator with an analytical precision of 0.2‰ (δ^15^N-N_2_O) and 0.4‰ (δ^18^O-N_2_O). All samples were analyzed alongside an internal N_2_O isotope standard that was previously calibrated at the University of Waterloo against local tropospheric air (assumed to be equal to 6.72‰ for δ^15^N and 44.62‰ for δ^18^O [17]). This internal standard gas was also submitted to UC Davis-SIF for isotopic analysis, and the standard deviation of 8 replicates (at varying concentrations including ambient) was 0.34‰ (for δ^15^N) and 0.77‰ (for δ^18^O). The absolute difference in the assigned value of this internal standard gas (blind inter-lab comparison) was 0.29‰ (for δ^15^N) and 0.81‰ (for δ^18^O). Given there are no internationally-recognized standardization methods or materials for N_2_O isotope analysis, these inter-laboratory results are in good agreement with one another. All values are reported here in units of per mill (‰) relative to air-N_2_ and VSMOW for δ^15^N and δ^18^O, respectively.

**Fig 2:**
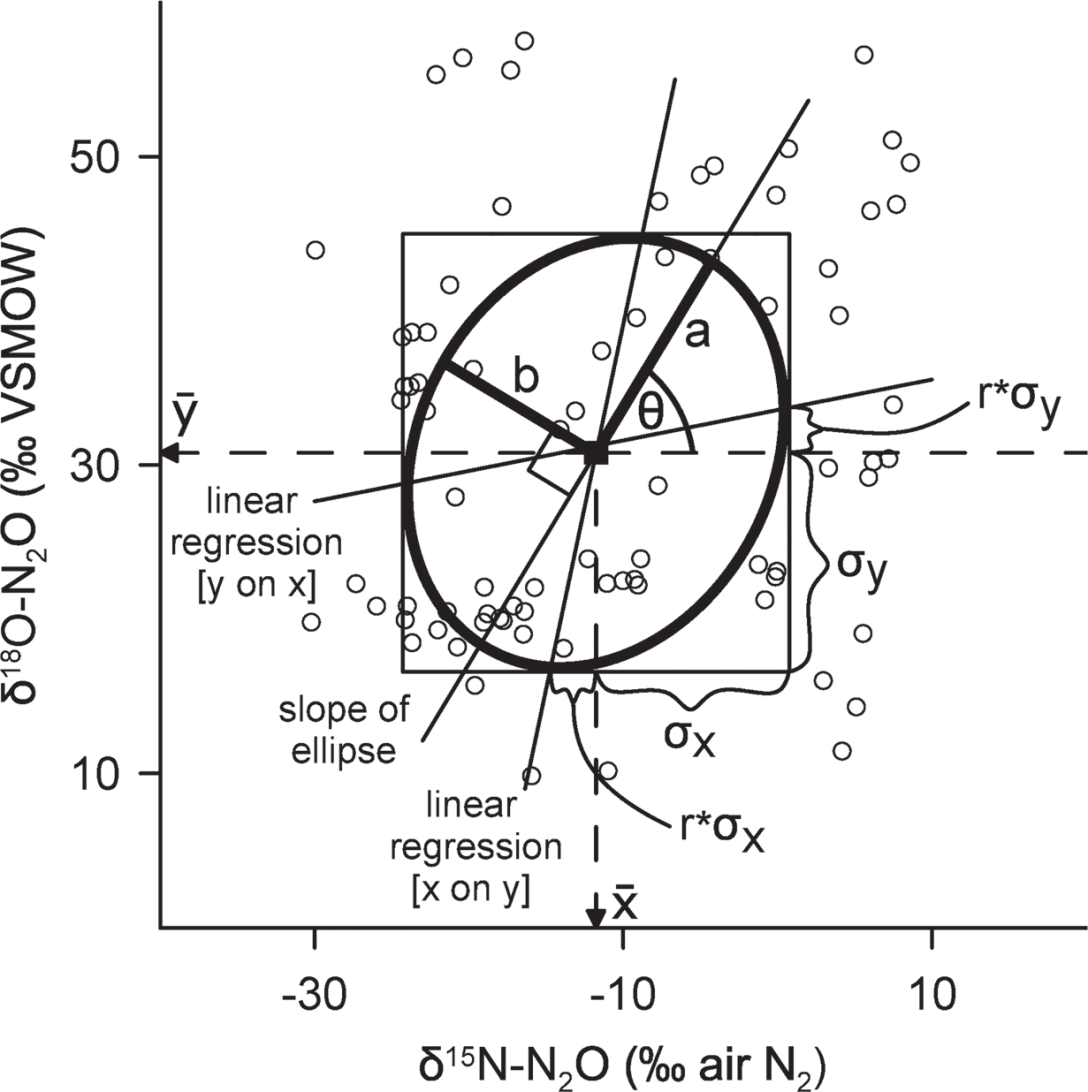
The standard ellipse of a bivariate sample. The urban wastewater N_2_O isotope data from Townsend-Small *et al*. [41], Toyoda *et al*. [65], and this study (*n* = 83) are summarized here with a standard ellipse. The centre of the ellipse is located at the sample mean (*x̄*, *ȳ*), where the semi-major (*a*) and semi-minor (*b*) axes intersect. The major axis is inclined versus the positive *x* axis by the angle *θ*. The tangent lines parallel to the *x* and *y* axes are related to the standard deviations (*σ*_*x*_, *σ*_*y*_) and the correlation coefficient (*r*). The two regression lines shown intersect the ellipse at the points of tangency [64].

All data were categorized as either Antarctic, freshwater, groundwater, marine, soil, stratosphere, troposphere, or urban wastewater, and a bivariate ellipse-based metric [63] was used to analyze and describe individual N_2_O reservoirs (Table 1). This circular statistical analysis is an improvement over other techniques that qualitatively summarize isotope data with a polygon or a freeform shape e.g., [6,15,46,47]. There is often a high degree of covariance between δ^15^N-N_2_O and δ^18^O-N_2_O and this statistical technique provides an accurate description of the central tendency of the data. By definition, the standard ellipse contains ∼40% of the data, is centered on the mean and has standard deviations of the bivariate data as semi-axes (Fig. 2) [63,64]. The data and an R file that contains the code to perform the statistical analyses and create the figures shown here are found at *https://github.com/jjvenky/Global-N2O-Ellipses*.

**Table 1:** Summary statistics of global δ^15^N-N_2_O and δ^18^O-N_2_O values as standard ellipses*.

Category	*n*	Sample-size-corrected ellipse area (‰ air N_2_ × ‰ VSMOW)	Mean δ^15^N ± 1σ (‰)	Mean δ^18^O ± 1σ (‰)	Correlation (*r*)	Semi-major axis	Semi-minor axis	Slope of ellipse	Theta (θ, rads)
Stratosphere	288	44	20.31 ± 20.79	56.39 ± 18.44	0.9994	27.8	0.5	0.89	0.73
Troposphere	225	0.47	6.55 ± 0.47	44.40 ±0.34	0.3758	0.5	0.3	0.46	0.43
Soil	884	296	−14.85 ± 12.01	31.23 ± 9.89	0.6083	14.0	6.7	0.73	0.63
Freshwater	738	203	−4.65 ± 9.84	41.77 ± 8.79	0.6656	12.1	5.4	0.85	0.70
Marine	495	92	6.63 ± 3.50	47.35 ± 9.54	0.4866	9.7	3.0	5.05	1.38
Groundwater	530	768	−13.97 ± 15.46	45.34 ± 17.74	0.4552	20.2	12.1	1.35	0.93
Antarctic	35	3086	−40.84 ± 30.75	29.03 ± 31.82	0.2256	34.7	27.9	1.16	0.86
Urban Wastewater	92	545	−11.56 ± 12.70	31.51 ± 14.14	0.2922	15.4	11.2	1.43	0.96

*A visual description of the standard ellipse is found in Fig. 2.

## Results and Discussion

**Fig 3:**
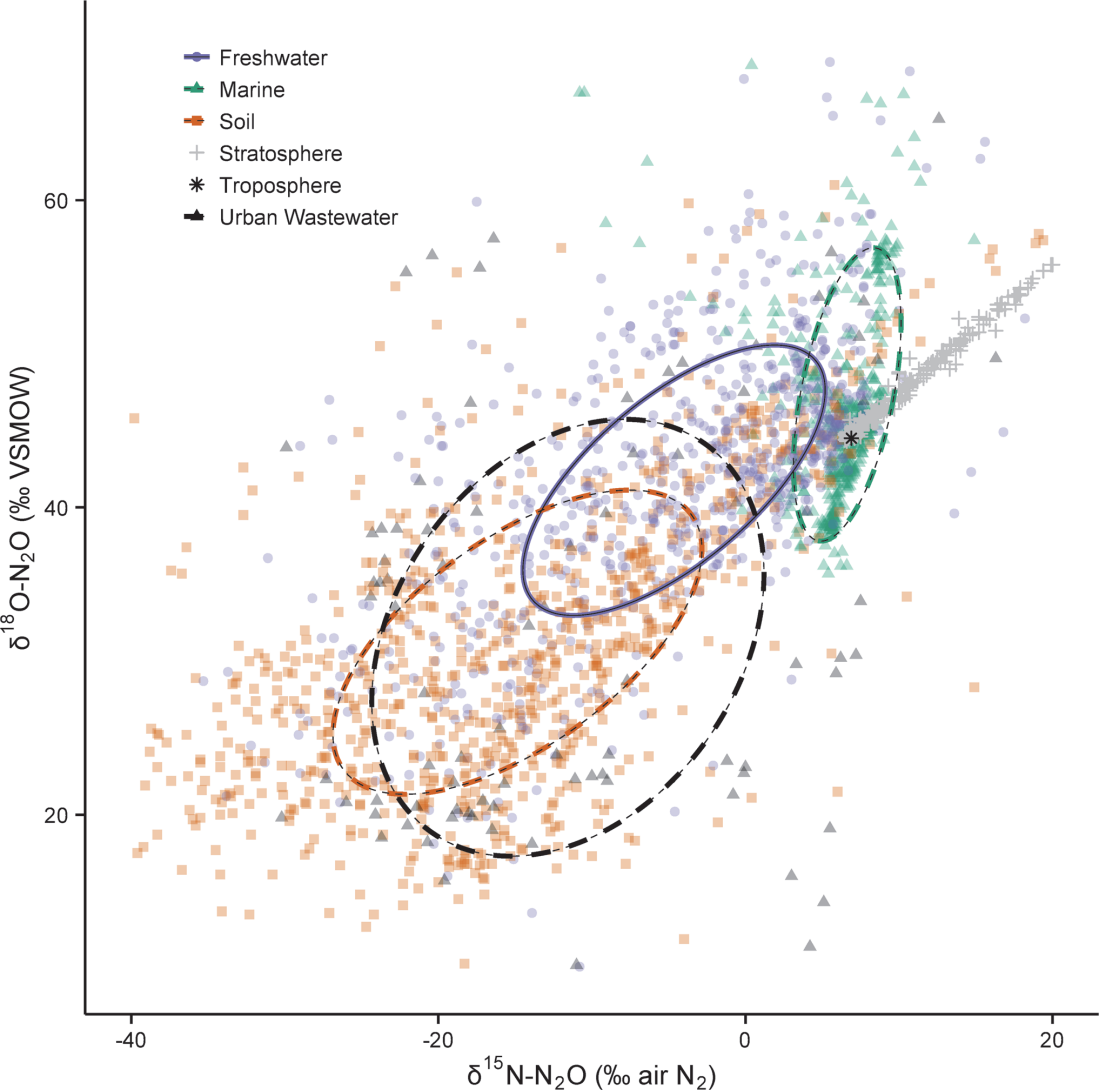
Global N_2_O isotope measurements from atmospheric samples and key environmental sources: freshwater, marine and soil (*n* = 2117). Data from municipal wastewater treatment plants is also included (*n* = 92). Each point represents one measurement, or in a few cases a reported average value. The colour of each point is two-thirds transparent to allow the density of data to be displayed. Although the ellipses are the same as in Fig. 1, the scales of the axes are narrowed to better show the data relative to current atmospheric values.

The data are highly non-uniform within and between categories (Figs. 1 and 3), and even within individual field sites (S1 Dataset). Historically, this has made it challenging to define an ‘isotopic signature’ for a given environment. Multiple factors cause this variability: (1) N_2_O is produced by nitrification and denitrification, and the isotopic composition of N and oxygen (O) endmembers can vary widely [26,31,32,54]; (2) the apparent fractionation of ^15^N/^14^N and ^18^O/^16^O during N transformations is not constant, nor is it easily predicted; and (3) oxygen exchange between N_2_O precursors and water imparts a large control on δ^18^O-N_2_O values during N_2_O formation. While the exact mechanisms are not fully understood, it appears that greater amounts of exchange occur in unsaturated environments than in saturated ones [66–68]. Additionally, the reduction of N_2_O to N_2_ in anaerobic environments causes enrichment of ^15^N and ^18^O isotopes in the remaining N_2_O pool, which displaces δ^15^N-N_2_O and δ^18^O-N_2_O values away from their original source values [26,51]. An initial analysis of all the data compiled in this study shows there is no clear separation of sources because each is described by an ellipse that overlaps at least one other source category (Figs. 1 and 3).

**Fig 4:**
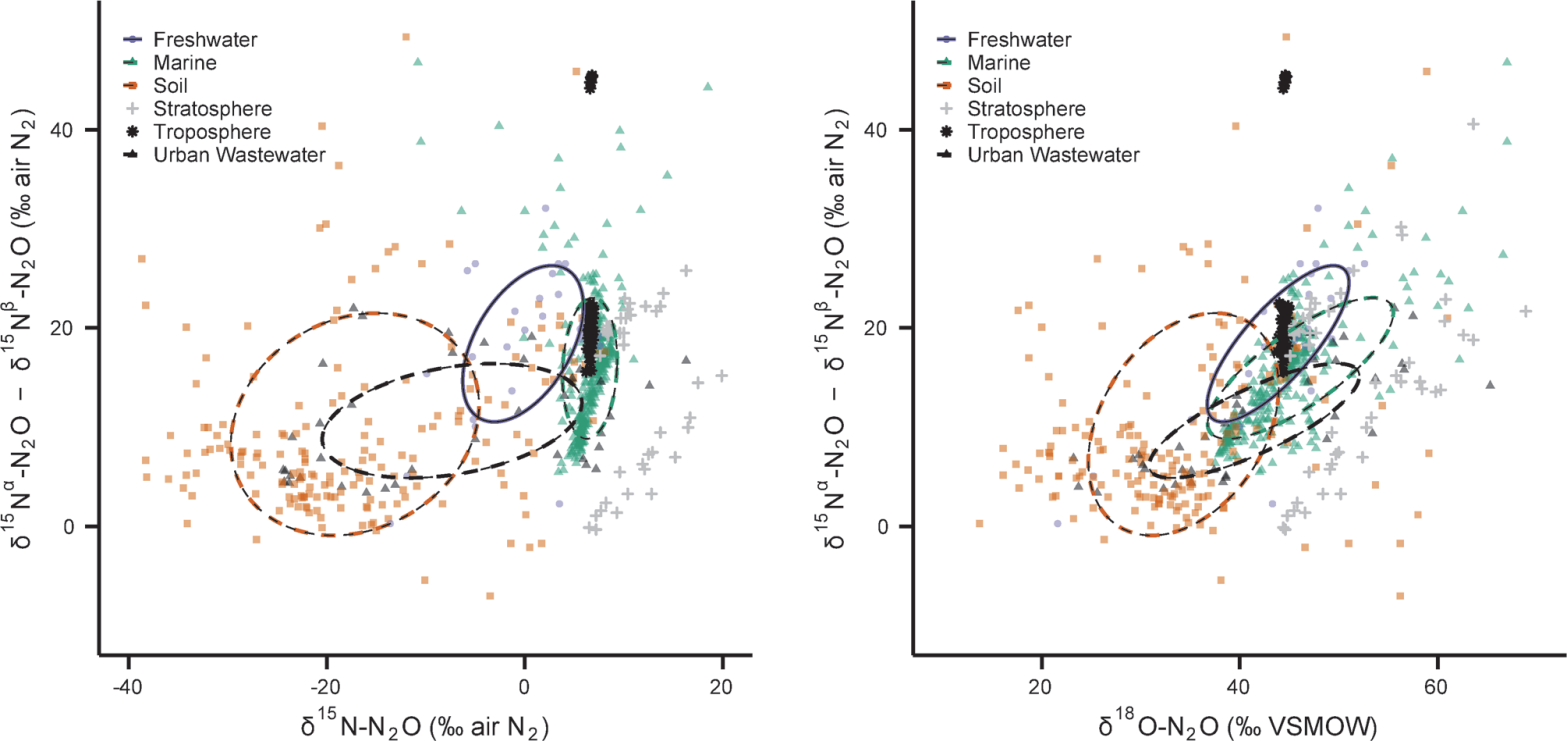
A comparison of ^15^N site preference values (*y*-axes) and (a) δ^15^N-N_2_O (left panel) and (b) δ^18^O-N_2_O (right panel) measurements from freshwaters, oceans, soils, atmosphere, and urban wastewater (*n* = 651). Measurements of ^15^N site preference from the Antarctic (*n* = 18) and groundwaters (*n* = 111) are not shown here, but are tabulated in S1 Dataset.

A similar comparison of all the published SP data (excluding Antarctic and groundwater categories) shows poor isotopic separation of sources (Fig. 4). It is difficult to determine how much of this variability is real, and how much is due to standardization issues and differences in measurement techniques. A recent inter-laboratory assessment of the methods used to determine nitrogen isotopomers revealed poor SP reproducibility [69]. Eleven laboratories employing either IRMS or laser spectroscopy techniques analyzed a single N_2_O target gas and the resulting standard deviation for SP was 4.24‰. Further, the inter-lab variation in the mean SP value was high, spanning a range of 11.62‰ [69]. This may help explain why there are two distinct groupings of SP data in each of the troposphere [2,17] and the stratosphere [29,45,58] (Fig. 4). Reaching an international consensus on standardization methods for the measurement and reporting of nitrogen isotopomers should vastly improve the utility of this data in source-apportionment studies at all scales. Finally, we note the reproducibility of δ^15^N-N_2_O and δ^18^O-N_2_O measurements in this recent round-robin test was much better than for SP, which gives us confidence in our ability to use these data here to make useful comparisons. The standard deviation (and range) of the N_2_O target gas in the inter-lab comparison was 1.37‰ (1.89‰) for δ^15^N-N_2_O and 1.00‰ (3.47‰) for δ^18^O-N_2_O [69].

**Fig 5:**
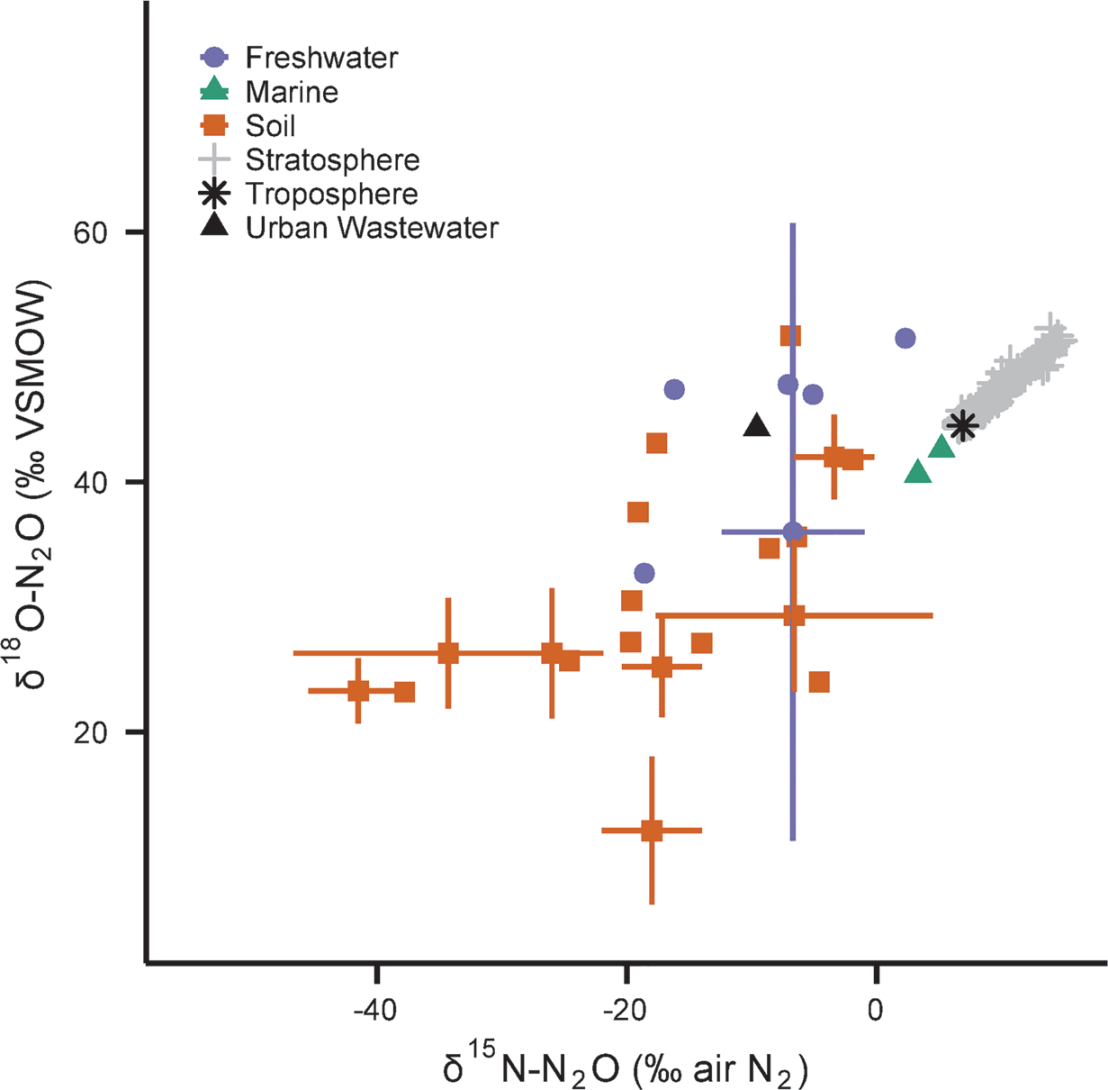
Emission-weighted average δ^15^N-N_2_O and δ^18^O-N_2_O from continental and marine environments. A small number of studies reported flux-weighted or flux and time-weighted average values. A few other studies provided information that allowed us to calculate these values. Equal weighting criteria were not applied in each case because not all values are time-weighted. Additional factors such as the sample size (*n*), antecedent conditions of N_2_O substrate(s), and time of year also different among studies (see S2 Dataset).

Much of the low concentration data from soils and surface waters are highly influenced by mixing with tropospheric N_2_O. This is evident by the high density of soil, freshwater and marine data that lies near the tropospheric N_2_O value (Fig. 3). In contrast, groundwater N_2_O, which does not mix with the atmosphere following recharge, is unaffected by this mixing process. Other processes such as substrate enrichment and N_2_O consumption control the isotopic composition of groundwater N_2_O, which displays extreme variability even within the same location (Fig. 1) [50,51]. Only 15 studies reported flux-weighted average δ^15^N-N_2_O, SP, and/or δ^18^O-N_2_O values, or provided enough information for us to calculate these values (2 freshwater studies, 2 marine studies, 10 soil studies, and 1 urban wastewater study; Fig. 5; S2 Dataset). The available flux-weighted data from soil and freshwater environments shows much overlap among this combined continental source, but the flux-weighted marine source appears to be unique. Importantly, there are very few flux-weighted data from all sources so robust conclusions cannot be made at this time. Additionally, these data were not weighted equally across studies so conclusions drawn from this analysis can be misleading. Only some of the values are time-weighted, and the sample size used to calculate the flux-weighted average varies from 3 to ∼50 (S2 Dataset). Emissions of N_2_O from soils (and potentially freshwaters and oceans) are inherently episodic, so future estimates of the flux-weighted average should attempt to include multiple measurements made over long timescales (months to years and encompassing seasonal differences) whenever possible.

**Fig 6:**
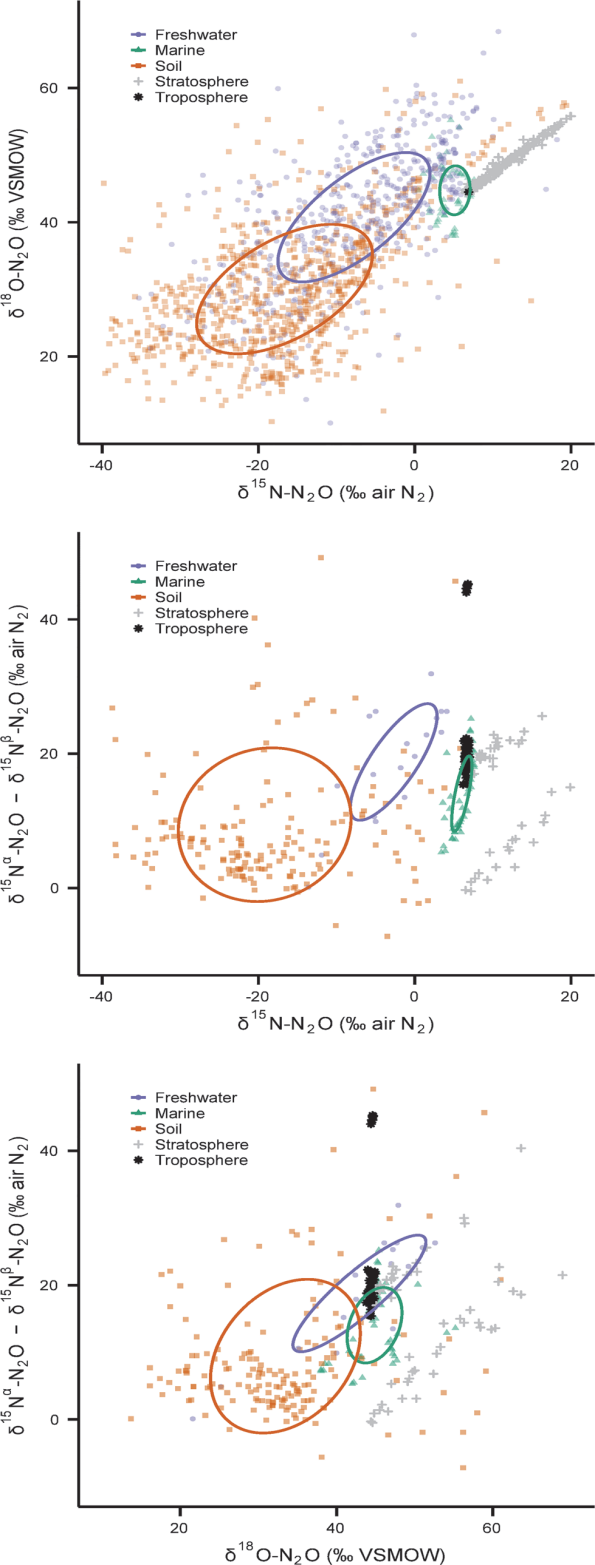
Nitrous oxide isotope measurements of key sources in the global isotope budget. Soil, freshwater and marine data were filtered to exclude samples that were highly influenced by mixing with tropospheric N_2_O: (a) δ^15^N-N_2_O vs. δ^18^O-N_2_O (top panel, *n* = 1383); (b) SP vs. δ^15^N-N_2_O (middle panel; *n* = 235); and (c) SP vs. δ^18^O-N_2_O (bottom panel; *n* = 235). The filtering criteria are described in the text.

The results of our analyses shown in Figs. 1 and 3 are not flux-weighted, nor are all the categories important atmospheric sources. For example, the most recent IPCC assessment reports that human excreta (all forms of treated/untreated sewage) contributes between 0.1–0.3 Tg N yr^−1^ as N_2_O, or only ∼1.1% of all natural and anthropogenic sources [5]. Of this, N_2_O emissions from urban wastewater treatment plants constitutes a very small fraction. To address this, we analyzed subsets of the data from important atmospheric sources (freshwaters, oceans, and soils) that were not strongly influenced by mixing with tropospheric N_2_O, and thereby make an important contribution to the flux-weighted average source value (Table 2; Fig. 6). To do this we filtered the data to include: (i) all reports of emitted N_2_O, regardless of the strength of the flux (two freshwater studies [13,46], two marine studies [16,43] and several soil studies) (see S2 Dataset); (ii) isotope data in the soil profile that had concentrations of N_2_O >650 ppb v/v (or 200% ambient); and (iii) isotope data in freshwater and near-surface marine environments (depths >100 m) with dissolved N_2_O concentrations >200% saturation with respect to atmospheric N_2_O [70].

**Table 2:** Summary statistics of filtered δ^15^N-N_2_O and δ^18^O-N_2_O data. These values show a subset of data that were not strongly influenced by mixing with tropospheric N_2_O, and thereby make an important contribution to the flux-weighted average source value (see Fig. 6).

Category	*n*	Sample-size-corrected ellipse area (‰ air N_2_ × ‰ VSMOW)	Mean δ^15^N ± 1σ (‰)	Mean δ^18^O ± 1σ (‰)	Correlation (*r*)	Semi-major axis	Semi-minor axis	Slope of ellipse	Theta (θ, rads)
Soil	794	288	−16.66 ± 11.24	30.05 ± 9.63	0.5341	13.0	7.0	0.75	0.64
Freshwater	527	215	−7.78 ± 9.72	40.75 ± 9.63	0.6821	12.5	5.5	0.99	0.78
Marine	62	22	5.14 ± 1.93	44.76 ± 3.62	0.0435	3.6	1.9	30.87	1.54
Continental*	1321	299	−13.11 ± 11.51	34.32 ± 10.96	0.6577	14.5	6.6	0.93	0.75

*The continental source, operationally defined here as Soil + Freshwater, is used along with the Marine source in our box-model calculations.

Most of the freshwater and soil data were retained (71% and 90%, respectively), and the ellipses of these data subsets are similar to the ellipses for all data in these categories (Table 1). Although the median δ^15^N and δ^18^O values of freshwater and soil N_2_O are significantly different (p < 0.001, Mann-Whitney test), their ellipses intersect one another at the 1σ level (Fig. 6–top panel), and we conclude that these sources are not isotopically distinct at the global scale. In order to further delineate freshwater and soil N_2_O more stable isotope measurements from freshwaters are needed; especially from non-temperate environments because the current data coverage from these systems is lacking. Measurements of SP may prove to be a useful means of separating these sources because the ellipses that describe SP vs. δ^15^N_bulk_ for freshwater and soils do not overlap (Fig. 6–middle panel).

Of the 495 published marine values compiled here, only 62 originated from the top 100 m of the ocean and were >200% saturation. Relative to continental N_2_O sources, the δ^15^N and δ^18^O values of near-surface oceanic N_2_O sources are poorly constrained. We found no reports of N_2_O isotope values from estuaries, which could represent an important fraction of the marine source but should be similar to marine or freshwater values. Tropical systems including reservoirs are also poorly studied. Future campaigns to more fully characterize δ^15^N and δ^18^O values of N_2_O emissions from aquatic environments, especially those impacted by anthropogenic N sources, are needed.

Although N_2_O generated from fossil fuel and biomass combustion may contribute ∼8% of the total source (or ∼20% of the anthropogenic source [5]), its isotopic composition is largely unknown. A lab-scale investigation of coal combustion revealed δ^15^N-N_2_O and δ^18^O-N_2_O values that were both enriched (unstaged combustion) and slightly depleted (air-staged combustion) relative to tropospheric N_2_O [71], indicating coal-derived N_2_O isotope values might be similar to marine sources but are dependent upon combustion conditions. A controlled study of gasoline-powered automobile exhaust concluded the average δ^15^N-N_2_O and δ^18^O-N_2_O values are similar to freshwater sources (−4.9 ±8.2‰ and +43.5 ± 13.9‰, respectively) [72]. Finally, N_2_O derived from biomass burning appears to closely resemble the δ^15^N and δ^18^O values of its endmembers; biomass-N and atmospheric O_2_, respectively [73]. We recognize the need to further investigate these potentially important sources and evaluate how they might affect the global N_2_O isotope budget.

**Fig 7:**
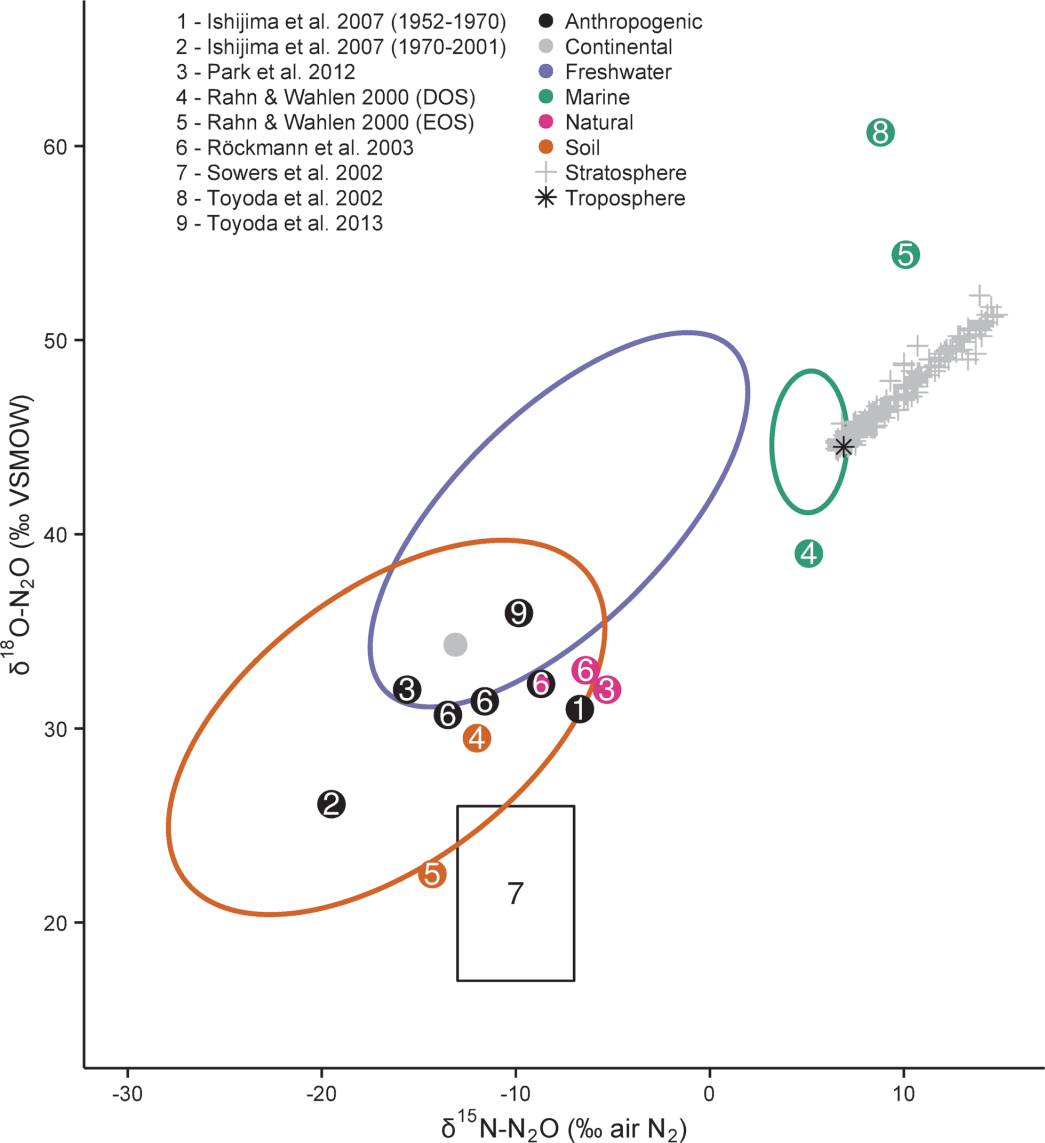
A comparison of bottom-up measurements to top-down estimates of N_2_O sources. Previous top-down studies have used a variety of modelling approaches to apportion the global N_2_O budget into different sources, identified here by colour. Ishijima *et al*. [10] measured N_2_O in firn air and calculated the isotopic composition of the anthropogenic source for two time periods: 1952‒1970 and 1970‒2001 that differed markedly in δ^15^N. Park et al. [2] constrained the pre-industrial, natural N_2_O source from δ^15^N-N_2_O and δ^18^O-N_2_O measurements in firn air and then calculated the current anthropogenic N_2_O source using recent archived air samples. Rahn and Wahlen [6] evaluated a depleted ocean scenario [DOS, originally proposed by Kim and Craig [20]], and an enriched ocean scenario [EOS, originally proposed by Kim and Craig [19]] to calculate corresponding terrestrial N_2_O sources. Röckmann *et al*. [9] measured N_2_O in firn air and modelled the pre-industrial (natural) source, the modern global average source (pink circle with black outline), and the anthropogenic source under the IPCC3 (higher value) and IPCC2 (lower value) scenarios. Sowers *et al*. [11] measured firn air and gas trapped in an ice core to calculate a range of values for the isotopic composition of the average anthropogenic N_2_O source. Toyoda *et al*. [7] estimated the δ^15^N and δ^18^O value of the oceanic N_2_O source using ‘Keeling Plots’ of detailed water column data. Toyoda *et al*. [8] monitored the isotopic ratio of tropospheric N_2_O in the northern hemisphere on a monthly basis from 2000–2011, and then used a box-model to estimate the current anthropogenic source. For reference, we show the Continental N_2_O source, which is used along with the Marine source in our box-model calculations, and is operationally defined as Soil + Freshwater.

After analyzing the filtered δ^15^N-N_2_O and δ^18^O-N_2_O data, the ‘bottom-up’ global N_2_O sources defined here were compared to estimates derived from ‘top-down’ atmospheric models (Fig. 7). Modelled estimates of the average anthropogenic and natural source fall within (or very close to) the soil ellipse *and* along a mixing line between soil and tropospheric N_2_O. All but two of the modelled estimates fall outside the freshwater ellipse, indicating the bulk of the combined anthropogenic and natural sources are not from freshwaters. If freshwaters were a major source of atmospheric N_2_O, a mixing line between freshwater and tropospheric N_2_O would be much closer to the anthropogenic and natural source values. It is not, and therefore we confirm what 'top-down' approaches have previously inferred: soil is the main source of N_2_O to the atmosphere.

We used the newly constrained δ^15^N-N_2_O and δ^18^O-N_2_O values for freshwater and soil (the combined continental source) and the ocean source to model their relative contributions to the total annual flux of N_2_O. We began with a box-model approach similar to that presented in [8,9,11,29]. We adapted the following isotope mass-balance equation for atmospheric N_2_O from Park *et al*. [29]:

$$Burden\times \frac{\partial \delta_{Trop}}{\partial t}=\sum Sources\left(\delta_{Sources}-\delta_{Trop}\right)-\left(\varepsilon \times L\right)$$ (1)

where, *Burden* = Present-day burden of N_2_O in the troposphere [1553 (± 21.742) Tg N] as reported by Stocker *et al*. [5]. This estimate is for year 2011, and is updated from data provided in Prather *et al*. [74], who report an uncertainty of 1.4% for year 2010.

$\frac{\partial \delta_{Trop}}{\partial t}$ = Deseasonalized, linear trend in archived samples of tropospheric N_2_O measured by Park *et al*. [2]. The linear trends for δ^15^N-N_2_O and δ^18^O-N_2_O are −0.035‰ yr^−1^ (± 0.002) and −0.022‰ yr^−1^ (± 0.004), respectively.

∑ *Source* = Annual N_2_O emissions from all sources (17.9 Tg N yr^−1^) [5]. For our calculations we applied an uncertainty of 25% to this parameter.

*δ*_*Sources*_ = Flux-weighted δ^15^N-N_2_O or δ^18^O-N_2_O value (‰) of the average modern source (all natural and anthropogenic sources).

*δ*_*Trop*_ = δ^15^N-N_2_O or δ^18^O-N_2_O value (‰) of the modern troposphere (provided in Table 1).

*ε* = Apparent enrichment factor (‰) for N_2_O destruction processes in the stratosphere. These values are taken from Table 3 in Park *et al*. [29], and are −14.9‰ (± 0.5) for ^15^N and −13.5‰ (± 0.5) for ^18^O. Note, the ratio of enrichment factors provided by Park *et al*. [29] (^18^O: ^15^N = 0.906) is very close to the slope of the regression line of the stratospheric N_2_O data shown in Fig. 1 (δ^18^O-N_2_O:δ^15^N-N_2_O = 0.886).

**Table 3:** MixSIAR model output summary.

	Category	Mean Contribution	Standard Deviation (σ)	Confidence Interval						
				2.5%	5%	25%	50%	75%	95%	97.5%
2-isotope (δ^15^N_bulk_, δ^18^O) mixing model	Soil	0.34	0.23	0.02	0.03	0.15	0.31	0.51	0.77	0.83
	Freshwater	0.24	0.16	0.01	0.02	0.11	0.22	0.35	0.51	0.57
	Marine	0.42	0.21	0.03	0.07	0.26	0.43	0.58	0.77	0.83
3-isotope (δ^15^N_bulk_, δ^18^O, SP) mixing model	Soil	0.33	0.22	0.02	0.03	0.15	0.31	0.49	0.72	0.78
	Freshwater	0.24	0.16	0.01	0.02	0.11	0.21	0.35	0.53	0.59
	Marine	0.43	0.19	0.07	0.13	0.30	0.42	0.55	0.75	0.82

*L* = Photochemical loss rate of N_2_O in the stratosphere (14.3 Tg N yr^−1^) [5]. Following [29], we applied an uncertainty of 25% in our calculations.

The term (− *ε* × *L*) is a very close approximation of the 'Net Isotope Flux' (‰ Tg N yr^−1^) as defined in [29], and is the net annual flux of N_2_O isotopologues from the stratosphere to the troposphere.

Equation 1 can be rearranged to solve for *δ*_*Sources*_ (Eq. 2), and all the known quantities provided above can be substituted into Eq. 2 to derive a flux-weighted, average modern source value (*δ*_*Sources*_) for δ^15^N-N_2_O and δ^18^O-N_2_O (‰).

$$\delta_{Sources}=\frac{\left(Burden\times \frac{\partial \delta_{Trop}}{\partial t}\right)+\left(\varepsilon \times L\right)+\left(\sum Sources\times \delta_{Trop}\right)}{\sum Sources}$$ (2)

Accordingly, we derive an average modern source value (± propagated standard deviation) for δ^15^N-N_2_O and δ^18^O-N_2_O of −8.4‰ (± 4.0) and +31.7‰ (± 13.9), respectively.

If we were to assume that all N_2_O fluxes *(F)* originate only from marine and continental sources, then:

$$\sum Sources\approx F_{Ocean}+F_{Cont}\approx 17.9\;{\text{Tg N yr}}^{-1}$$ (3)

and the flux-weighted modern source value is approximated by:

$$\delta_{Sources}\approx \frac{\left(\delta_{Cont}\times F_{Cont}\right)+\left(\delta_{Ocean}\times F_{Ocean}\right)}{\sum Sources}$$ (4)

where the δ value of the continental and ocean sources are given in Table 2.

Combining Eq. 2 with Eq. 4 yields:

$$\left(\delta_{Cont}\times F_{Cont}\right)+\left(\delta_{Ocean}\times F_{Ocean}\right)\approx \left(Burden\times \frac{\partial \delta_{Trop}}{\partial t}\right)+\left(\varepsilon \times L\right)+\left(\sum Sources\times \delta_{Trop}\right)$$ (5)

Given the assumption that *F_Cont_* ≈ ∑ *Sources* − *F_Ocean_* (Eq. 3), we can approximate *F*_*ocean*_ by:

$$F_{Ocean}\approx \frac{\left(Burden\times \frac{\partial \delta_{Trop}}{\partial t}\right)+\left(\varepsilon \times L\right)+\sum Sources\left(\delta_{Trop}-\delta_{Cont}\right)}{\delta_{Ocean}-\delta_{Cont}}$$ (6)

Accordingly, using N isotope ratios we derive a value for *F*_*ocean*_ of ∼4.6 (± 12.6) Tg N yr^−1^, which is ∼26% of all sources (17.9 Tg N yr^−1^) [5]. The *F*_*Cont*_ is found by difference, and is approximately equal to 13.3 (± 13.4) Tg N yr^−1^, or 74% of all natural and anthropogenic N_2_O sources. The largest source of uncertainty in the N isotope mass-balance lies in the δ^15^N value of the continental source (1σ = 11.5‰), followed by ∑ *Sources* and *L*, which have a relative uncertainty of 25% in our model.

The most recent N_2_O budget estimates the combined soil, freshwater, and ocean flux to be ∼15.7 Tg N yr^−1^, or 87.7% of the total source [5]. Our approach assumes the ∑ *Sources* = 17.9 Tg N yr^−1^ (as reported in IPCC-AR5) because other terms in the mass-balance (e.g., N_2_O burden and loss rate) are based on a budget that includes all known sources. As such, we ignore the contributions from smaller sources such as human sewage, fossil fuels, industry, biomass combustion, and chemical production processes in the atmosphere, which have a combined annual flux of ∼2.2 Tg N yr^−1^ [5]. Despite this, our result for *F*_*ocean*_ is similar to the estimate provided in IPCC-AR5, which shows oceans contribute 21% of the annual N_2_O budget [5].

The O isotope mass-balance fails to derive a positive ocean flux (*F*_*ocean*_ = −4.5 ± 19.9 Tg N yr^−1^). This is because the δ^18^O separation between the troposphere and the continental source is smaller than it is for δ^15^N, and the term ∑ *Sources* (*δ*_*Trop*_ − *δ*_*Cont*_) is too small to make the numerator in Eq. 6 a net positive number. However, decreasing the loss rate (*L*) by 4 Tg N yr^−1^ and increasing ∑ *Sources* by the same amount yields a positive ocean flux = 4.6 Tg N yr^−1^. Therefore, if the uncertainty of these parameters is reduced in the future we may find that the O isotope budget balances.

Finally, we used a stable isotope Bayesian mixing model (MixSIAR) [75] to determine the proportions of soil, freshwater, and marine N_2_O that best predicted the average modern source (anthropogenic plus natural) (Eq. 7).

$$\delta_{Sources}=\left(\delta_{Soil}\times F_{Soil}\right)+\left(\delta_{Freshwater}\times F_{Freshwater}\right)+\left(\delta_{Ocean}\times F_{Ocean}\right)$$ (7)

where, *F_Soil_* + *F_Freshwater_* + *F_Ocean_* = 1.

MixSIAR, which is a front-end interface of the model SIAR (Stable Isotope Analysis in R) [76], is an ecological mixing model traditionally used to describe food web and predator-prey relationships. Values of δ^15^N_bulk_-N_2_O, δ^15^N^α^-N_2_O, δ^15^N^β^-N_2_O, and δ^18^O-N_2_O for the average modern source (the mixture) were taken from Röckmann *et al*. [9]. These estimates are almost identical to the ones calculated in our 2-box-model (above), and Röckmann *et al*. [9] provided ^15^N isotopomers, which allowed us to use 3 variables in our model runs (δ^15^N_bulk_, δ^18^O, and SP). A series of Markov Chain Monte Carlo simulations, using values for soil, freshwater, and marine N_2_O (filtered, raw data compiled in this study), were done to find mixing solutions that best fit the average modern source (Table 3). Gelman-Rubin and Geweke diagnostic tests indicated a chain length of 300,000, burn in of 200,000, thinning of 50 (2-isotope) or 100 (3-isotope), and 3 chains were appropriate.

Model runs using only the δ^15^N-N_2_O and δ^18^O-N_2_O data (2-isotope mixing model, *n* = 1383 data pairs) produced results very similar to the model runs that also included SP data (3-isotope mixing model, n = 235 data triads). Overall, this Bayesian modeling exercise predicted the soil, freshwater, and ocean contributions (± 1σ) to the average modern N_2_O source were 0.43 (0.20), 0.34 (0.22), and 0.24 (0.16), respectively (Table 3). Unlike the box-model, this approach does not place *a priori* bounds on the data, and is not constrained by terms such as the stratospheric N_2_O loss rate (*L*), which have large uncertainty. However, this method also ignores the contributions of several small sources, which have a combined contribution of ∼12.3% to the total budget presented in IPCC-AR5 [5].

Both SIAR and the box-model predict the ocean flux to be 24% and 26% of the total, respectively, which closely confirms the scientific community's best estimate of the ocean flux as presented in IPCC-AR5 (21% of the total source). Further, the SIAR model output shows that freshwaters may contribute much more N_2_O than previously thought. The current N_2_O budget estimates the combined flux from rivers, estuaries, and coastal zones is 0.6 Tg N yr^−1^, or just 3% of the total source. While we acknowledge that there is δ^15^N-δ^18^O overlap in the soil and freshwater source (Fig. 6–top and bottom panels), these sources appear to be unique in δ^15^N-SP space (Fig. 6–middle panel). Therefore, we suggest there is a great need to quantify N_2_O fluxes from freshwaters, estuaries, and coastal zones, which have received considerably less attention than soil and off-shore marine environments.

## Supporting Information

S1 Dataset A comma-delimited text file with all the data collected and analyzed in this study. In addition to δ^15^N-N_2_O, SP and δ^18^O-N_2_O values, we provide a reference citation, the category, a brief site description, and the criteria used to filter the data subsets (S1_Dataset.csv).
(CSV)

S2 Dataset A comma-delimited text file with weighted average δ^15^N-N_2_O, SP and δ^18^O-N_2_O values from select freshwater, soil and urban wastewater studies (S2_Dataset.csv). (CSV)
