# Correction: From the Ground Up: Global Nitrous Oxide Sources are Constrained by Stable Isotope Values

**DOI:** 10.1371/journal.pone.0149290

**Published:** 2016-02-09

**Authors:** David M. Snider, Jason J. Venkiteswaran, Sherry L. Schiff, John Spoelstra

There are errors in Table 3. The labels under the “Category” column are incorrect. Please see the corrected Table 3 here.

**Table 3:** MixSIAR model output summary.

				Confidence Interval						
	Category	Mean Contribution	Standard Deviation (σ)	2.5%	5%	25%	50%	75%	95%	97.5%
	Freshwater	0.34	0.23	0.02	0.03	0.15	0.31	0.51	0.77	0.83
2-isotope (δ^15^N_bulk_, δ^18^O) mixing model	Marine	0.24	0.16	0.01	0.02	0.11	0.22	0.35	0.51	0.57
	Soil	0.42	0.21	0.03	0.07	0.26	0.43	0.58	0.77	0.83
	Freshwater	0.33	0.22	0.02	0.03	0.15	0.31	0.49	0.72	0.78
3-isotope (δ^15^N_bulk_, δ^18^O, SP) mixing model	Marine	0.24	0.16	0.01	0.02	0.11	0.21	0.35	0.53	0.59
	Soil	0.43	0.19	0.07	0.13	0.30	0.42	0.55	0.75	0.82

## References

[1] Snider DM, Venkiteswaran JJ, Schiff SL, Spoelstra J (2015) From the Ground Up: Global Nitrous Oxide Sources are Constrained by Stable Isotope Values. PLoS ONE 10(3): e0118954 doi:10.1371/journal.pone.0118954 25811179 10.1371/journal.pone.0118954 PMC4374930

